# Targeted radiotherapy of brain tumours

**DOI:** 10.1038/sj.bjc.6601771

**Published:** 2004-04-06

**Authors:** M R Zalutsky

**Affiliations:** 1 Department of Radiology, Duke University Medical Center, PO Box 3808, Durham, NC 27710, USA

**Keywords:** glioblastoma multiforme, radiotherapy, radioimmunotherapy, glioma, anaplastic astrocytoma

## Abstract

The utility of external beam radiotherapy for the treatment of malignant brain tumours is compromised by the need to avoid excessive radiation damage to normal CNS tissues. This review describes the current status of targeted radiotherapy, an alternative strategy for brain tumour treatment that offers the exciting prospect of increasing the specificity of tumour cell irradiation.

Even with aggressive multi-modality treatment strategies, the life expectancy for patients with glioblastoma multiforme (GBM), the most common and virulent primary brain tumour, is less than a year from the time of diagnosis (Stewart, 2002). The vast majority of glioma patients experience local recurrence, with a median survival of only 16–24 weeks for those with recurrent disease (Wong *et al*, 1999). Conventional radiotherapy continues to play a primary role in brain cancer treatment; however, its lack of tumour specificity is a severe limitation of this form of therapy. Owing to its nonspecific nature, toxicity to normal brain limits the radiation dose that can be delivered to tumour cells, and compromises the quality of life of the few longer-term survivors.

An emerging approach for brain tumour treatment is targeted radiotherapy, a strategy that utilises a molecular vehicle to selectively deliver a radionuclide to malignant cell populations. An important consideration is to match the decay properties of the radionuclide with the characteristics of the tumour. The two most commonly utilised radionuclides in targeted radiotherapy, ^131^I and ^90^Y, emit beta particles, which deposit 95% of their energy within 0.992 and 5.94 mm, respectively (Hopkins *et al*, 1998). Radiation of this type might be best suited for treating tumour that may be present in the 2-cm rim around the brain tumour resection cavity, where most gliomas recur. On the other hand, alpha particles have a tissue range of only a few cell diameters and might be ideal for elimination of the small clusters or single glioma cells that can occur 4–7 cm from the primary tumour site (Goldbrunner *et al*, 1999). Alpha particles could potentially enhance the therapeutic potential for minimal residual disease by maximising the radiation dose received by these relatively small tumour foci, while sparing normal CNS tissues.

Although many types of labelled molecules have been explored for targeted cancer radiotherapy, investigations in brain tumour patients have almost exclusively utilised monoclonal antibodies (mAbs) as the targeting vehicle. Brain-tumour-associated molecular targets that have been evaluated for radioimmunotherapy include the epidermal growth factor receptor (Brady *et al*, 1992) and the human neural cell adhesion molecule (NCAM), which is present both on glioma as well as normal neural tissue (Hopkins *et al*, 1998). However, the vast majority of targeted radiotherapy studies in brain tumour patients have utilised radiolabelled mAbs reactive with the tenascin molecule (Table 1

**Table 1:** Summary of targeted radiotherapy clinical trials in brain tumour patients

**Radiotherapeutic**	**Design**	**Patients^a^**	**Toxicities^b^**	**Survival (weeks)**	**Reference**
I-131 81C6 murine mAb	Phase I	34 (26 GBM) recurrent	Neuro Heme	60 (56 GBM)	Bigner *et al* (1998)
I-131 81C6 murine mAb	Phase I	42 (32 GBM) newly diagnosed	Neuro	79 (69 GBM)	Cokgor *et al* (2000)
I-131 81C6 murine mAb	Phase II	33 (27 GBM) newly diagnosed	Neuro Heme	87 (79 GBM)	Reardon *et al* (2002)
At-211 81C6 chimeric mAb	Phase I	17 (14 GBM) recurrent	None	60	Zalutsky *et al* (2002)
I-131 BC-2 or BC-4 mAb	Phase II	91 (74 GBM)	None	>184 AA	Riva *et al* (2000)
3–10 cycles		44 recurrent		76 GBM	
		47 newly diagnosed			
Y-90 BC-2 or BC-4 mAb	Phase II	43 (35 GBM)	None	360 AA	Riva *et al* (2000)
3–5 cycles				80 GBM	
Y-90 or I-131 BC-4 mAb	Phase II	37 (24 GBM)	Neuro	68 GBM	Goetz *et al* (2003)
Mean, three cycles					
BC-4 mAb, Y-90 biotin	Phase I	24 (16 GBM) recurrent	Neuro	76 AA	Paganelli *et al* (2001)
two cycles				50 GBM	
BC-4 mAb, Y-90 biotin	Phase II	8 GBM	None	134	Grana *et al* (2002)
two cycles					

a GBM=glioblastoma multiforme; AA=anaplastic astrocytoma.

b Neuro=neurological toxicity; Heme=haemtatological toxicity.

).

## TENASCIN AND ANTI-TENASCIN MABS

Tenascin-C is a hexabrachion polymorphic glycoprotein that is overexpressed in the extracellular matrix in high-grade gliomas as well as other tumour types. The expression of tenascin increases with advancing tumour grade, with more than 90% of glioblastoma multiforme biopsies exhibiting very high levels of tenascin expression. Tenascin-C expression occurs primarily around tumour-supplying blood vessels, with this staining pattern becoming more pronounced with increasing tumour grade (Herold-Mende *et al*, 2002). Furthermore, in WHO II and III gliomas, there appears to be a correlation between perivascular staining and earlier tumour recurrence. The mAb BC-4 binds to an epitope within the epidermal growth factor (EGF)-like repeat found on all tenascin isoforms, while BC-2 reacts with an epitope found on the alternatively spliced fibronectin type III repeats A1 and A4, which share 83% homology (Balza *et al*, 1993). The anti-tenascin mAb developed by our group, 81C6 (Bourdon *et al*, 1983), binds to an epitope within the alternatively spliced fibronectin type III CD region. In the following sections, the current status of targeted brain tumour radiotherapy with these mAbs, both directly labelled and as part of a pretargeting strategy, will be reviewed.

## ANTI-TENASCIN MAB 81C6

In order to exploit the potential utility of tenascin as a molecular target for radioimmunotherapy, mAb 81C6, a murine IgG_2b_, was developed. The ability of murine 81C6 to selectively localise and treat human glioma xenografts was investigated extensively in rodent models before the initiation of clinical studies with this labelled mAb (Wikstrand *et al*, 2001). Three observations from diagnostic-level investigations performed in glioma patients had an influence on the design of our subsequent targeted radiotherapy protocols. First, levels of ^131^I-labelled 81C6 in tumour biopsies were up to five times higher than a co-injected ^125^I-labelled nonspecific mAb, and up to 200 times higher than those in normal brain (Zalutsky *et al*, 1989). Thus, within the brain, uptake of ^131^I-labelled 81C6 was both selective and specific. Second, intracarotid injection did not significantly increase tumour delivery compared with intravenous administration (Zalutsky *et al*, 1990). And third, an mAb protein escalation study demonstrated that intravenous administration could not be used to deliver therapeutically useful levels of labeled mAb to tumour without excessive dose to liver and spleen, normal organs that express tenascin (Schold *et al*, 1993). This led us to focus on a compartmental approach involving direct administration of radiolabelled 81C6 mAb into surgically created tumour resection cavities.

### Iodine-131-labelled 81C6 clinical trials

Our clinical experience with ^131^I-labelled 81C6 mAb currently includes over 300 patients who have received the labelled mAb by direct injection into a surgically created resection cavity via a Rickham reservoir and catheter placed at the time of resection. The entry criteria for parallel phase I studies in patients with recurrent and newly diagnosed malignant glioma included: histopathological confirmation of diagnosis, demonstration of tumour reactivity with 81C6 by immunohistochemistry, a maximum of 1 cm residual enhancement on postoperative MRI, and tumor localisation within the supratentorial compartment. Patency of the catheter and intactness of the resection cavity was confirmed by radionuclide imaging prior to treatment, and patients received a saturated solution of potassium iodide to block thyroid uptake of radioiodine that might be released from the labelled mAb. Most patients received systemic chemotherapy after the radioimmunotherapy procedure, and newly diagnosed patients also received external beam radiotherapy.

The phase I study of recurrent disease enrolled 34 patients, including 26 with GBM (Bigner *et al*, 1998). The administered dose of ^131^I-labelled 81C6 ranged from 740 to 4440 MBq (20–120 mCi), and a maximum tolerated dose of 3700 MBq (100 mCi) was established. Dose-limiting toxicity was neurologic. The median survival for patients with recurrent GBM and for all patients treated was 56 and 60 weeks, respectively. In the parallel phase I study in patients with newly diagnosed brain tumours, a total of 42 patients were treated including 32 with glioblastoma multiforme (Cokgor *et al*, 2000). The administered activity of ^131^I-labelled 81C6 ranged from 740 to 6660 MBq (20–180 mCi). A maximum tolerated dose of 4440 MBq (120 mCi) was established, with dose-limiting toxicity again being neurologic. Reversible haematologic toxicity was observed in seven patients. Patient-specific dosimetry calculations indicated that the 2-cm thick region surrounding the resection cavity interface received an average radiation dose of 32 Gy (range 2–59 Gy) (Akabani *et al*, 2000). The median survival for patients with GBM and all patients was highly encouraging at 69 and 79 weeks, respectively.

A phase II trial was then conducted in patients with newly diagnosed malignant glioma at an administered activity of 4440 MBq (120 mCi) of ^131^I-labelled murine 81C6 (Reardon *et al*, 2002). In all, 33 patients were enrolled including 27 with GBM. Treatment-related neurotoxicity was observed in five patients and reversible haematologic toxicity occurred in nine patients. It should be noted that irreversible neurotoxicity was associated with the resection cavity being either contiguous or adjacent to the compromised CNS functional centre. The median survival achieved for patients with GBM and all patients enrolled in this trial was 79 and 87 weeks, respectively. These results were compared to those predicted by a recursive partitioning model (Curran *et al*, 1993). For example, this model predicted a median survival of 55 weeks for newly diagnosed GBM patients less than 50 years old, while in our study an 87-week median survival was achieved. Likewise, newly diagnosed GBM patients over 50 years old with a Karnofsky performance status greater than 70% were predicted to have a median survival of 39 weeks compared with 65 weeks for those in that category in our study.

Other methodologies such as stereotactic radiosurgery and ^125^I interstitial brachytherapy also have been investigated for delivering a boost radiation dose to tumour, and the prolonged survival achieved in this phase II study compared favourably to these approaches. However, an important distinction between targeted radiotherapy with ^131^I-labelled 81C6 and these other techniques was with regard to the need for re-operation to debulk radiation necrosis and to relieve symptomatic mass effect. Re-operation rates for brachytherapy and radiosurgery have been reported to be in the 30–60% range, while only 2% of the 109 combined patients on our phase I and II trials required re-operation for symptomatic radionecrosis.

Owing to regulatory constraints, radioiodinated mAb was given based on ^131^I activity rather than on a calculated radiation dose to the resection cavity margins. Dosimetry calculations indicated that this critical parameter could vary considerably due to differences in the rate of clearance of radioactivity from the resection cavity and the resection cavity volume. In the 42 patients entered on the phase I newly diagnosed trial, radionuclide residence times in the cavity ranged from 10 to 113 h, and cavity volumes from 2 to 81 cm^3^ (Akabani *et al*, 2000). This led to average radiation dose deposited in the 2-cm resection cavity margin ranging from 3 to 59 Gy.

A 16-patient subset of these newly diagnosed glioma patients experienced progressive changes on serial MRI images and had biopsies, permitting an investigation of the relationship between histopathology and the radiation dose delivered to the 2-cm cavity margin. Patients receiving a dose less than 44 Gy generally had tumour recurrence, while those receiving more than 44 Gy to the 2-cm margin had a greater incidence of radiation necrosis. In an attempt to maximise local tumour control while minimising normal brain radionecrosis, we have now initiated another phase I study in which newly diagnosed patients are given an activity level of ^131^I-labelled 81C6, based on a prior dosimetry study, calculated to deliver an average of 44 Gy to the resection cavity margins. To date, 17 patients have been treated on this protocol; however, the median follow up period is not sufficient to provide meaningful survival data.

### Astatine-211 labelled chimeric 81C6 clinical trial

An important variable for targeted brain tumour radiotherapy is the nature of the radiation emitted by the radionuclide. Although use of molecules labelled with ^131^I or other beta emitters can increase the selectivity of tumour cell irradiation compared with external beam irradiation, both approaches have similar radiobiological effectiveness. An advantage of alpha particles such as those emitted by the 7.2-h half-live radiohalogen ^211^ At is that they are high linear energy transfer (LET) radiation, with considerably higher cytotoxicity. Cell culture experiments have demonstrated that human tumour cell lines could be killed with only a few alpha particle traversals per cell (Zalutsky and Vaidyanathan, 2000). Furthermore, the cytotoxicity of alpha particles is nearly independent of dose rate, oxygen concentration and cell cycle stage. As tenascin expression is perivascular, combining anti-tenascin mAb 81C6 with a radionuclide that emits short-range radiation might be a particularly effective approach, in that it could act as a vascular targeted therapeutic (Akabani *et al*, 2002). In this way, it might be possible to also kill tumour cells indirectly by compromising their blood supply.

A phase I trial is currently being performed to determine the maximum tolerated dose, pharmacokinetics and objective responses to ^211^At-labelled chimeric 81C6 administered into surgically created glioma resection cavities in recurrent glioma patients (Zalutsky *et al*, 2002). The chimeric construct has human IgG2 constant region domains and was selected for this protocol because its stability *in vivo* was shown to be considerably higher than that of murine 81C6 (Reist *et al*, 1998). The ^211^At was produced at the Duke University Medical Center cyclotron and the mAb was labelled with preservation of immunoreactivity by reaction with *N*-succinimidyl 3-[^211^At]astatobenzoate. To date, 17 patients (three anaplastic oligodendroglioma, 14 glioblastoma multiforme) have received 10 mg of mAb labelled with escalating activities (74 MBq, *n*=5; 148 MBq, *n*=6; 248 MBq, *n*=5; 370 MBq, *n*=1) of ^211^At-labelled chimeric 81C6. As this was the first clinical trial of any ^211^At-labelled radiotherapeutic, demonstration of *in vivo* stability and safety were particularly important. Serial blood counting and imaging were performed and indicated very low levels of leakage of ^211^At from the surgical resection cavity. Less than 0.2% of the injected dose was found in the blood pool and more than 95% of the ^211^At decays occurred within the tumour resection cavity. Cavity interface radiation doses were in the range of 150–35 000 Gy (2986 Gy average dose) compared with 0.01 Gy for normal organs including tenascin-expressing spleen and liver. Encouraging responses have been obtained with a median survival of 60 weeks observed in all patients. Particularly encouraging is the fact that two patients with recurrent GBM survived for more than 150 weeks, and a patient with recurrent anaplastic oligodendroglioma is now approximately 215 weeks from treatment. The maximum tolerated dose of ^211^At-labelled chimeric 81C6 has yet to be defined.

## STUDIES WITH BC-2 AND BC-4 ANTI-TENASCIN MABS

(Riva *et al*, 2000) have been evaluating the efficacy of ^131^I-labelled and ^90^Y-labelled BC-2 and BC-4 mAbs for the locoregional treatment of malignant gliomas. In these protocols, no distinction was made between the two mAbs. The phase II study with ^131^I involved 91 patients including 74 with glioblastoma and nine with anaplastic astrocytoma. At the time of treatment, 52 patients were classified as having small (less than 2 cm^3^) or undetectable residual tumour, with the remainder having a larger tumour mass. The study population consisted of 47 newly diagnosed and 44 recurrent tumours. Patients received three to 10 cycles of ^131^I-labelled mAb, at intervals of either 1 or 3 months, with a cumulative administered activity of up to 20.35 GBq (550 mCi). The median survival was >46 months in anaplastic astrocytoma and 19 months in glioblastoma, with no distinction made between newly diagnosed and recurrent patients groups. The response rate in glioblastoma patients was better in those with small volume (56.7%) compared with larger tumours (17.8%).

A subsequent study was performed using ^90^Y in order to investigate the potential effects of using a radionuclide emitting beta particles with greater tissue penetration (Riva *et al*, 2000). In this phase II investigation, 43 patients were treated, including six with anaplastic astrocytoma and 35 with glioblastoma. In all, 16 were classified as having small volume or minimal disease at the time of treatment. Patients received between three and five cycles of ^90^Y-labelled mAbs with a cumulative activity of up to 3.145 GBq (85 mCi). The median survival for patients with anaplastic astrocytoma and glioblastoma was 90 months and 20 months, respectively. The response rate in glioblastoma patients was 26.3% in those with bulky disease compared with 56.3% for those with smaller lesions.

In a more recent study, the therapeutic potential of ^131^I- and ^90^Y-labelled BC-4 mAb were evaluated in 37 patients, consisting of 13 with astrocytoma WHO grade III and 24 with WHO grade IV histology (Goetz *et al*, 2003). Multiple cycles of labelled mAbs were administered (mean, three per patient) at various activity levels. The median survival for glioblastoma patients was 17 months. No attempt was made to stratify analyses according to the radionuclide used or whether the patients had recurrent or newly diagnosed lesions.

These clinical studies are important in that they confirm the potential of locoregionally administered labelled mAbs as a means for improving the survival of patients with malignant brain tumours. The low incidence of side effects, even after multiple cycles, also is encouraging. However, it remains to be ascertained whether use of the higher energy beta emitter ^90^Y and multiple cycles of labelled mAb results in a significant improvement in therapeutic efficacy compared with a single dose of ^131^I-labelled mAb.

## PRETARGETED RADIOIMMUNOTHERAPY

One of the limitations of directly labelled mAbs for targeted radiotherapy is that as a consequence of their macromolecular size they diffuse slowly through tissue, hampering their delivery to tumour cells distant from their site of injection. An attractive strategy to compensate for the large size of mAbs is pretargeting, a procedure in which the mAb is administered first, followed after an appropriate time interval by the injection of a radiolabelled low molecular weight vehicle. The most common approach attempts to exploit the extraordinarily high affinity of avidin or streptavidin for the 244 Da vitamin, biotin. A three-step avidin-biotin based regimen has been investigated in glioma patients who first received biotinylated BC-4 mAb, followed 24 h later by avidin, and finally, after an additional 18 h, a ^90^Y-labelled biotin conjugate.

The first trial with this pretargeting approach was performed in patients with recurrent glioma and the three reagents were administered via a catheter placed into the surgical resection cavity (Paganelli *et al*, 2001). In all, 16 patients with glioblastoma and eight with anaplastic astrocytoma were treated with two cycles administered 8–10 weeks apart. The maximum tolerated dose was 1.11 GBq (30 mCi) of ^90^Y-labelled DOTA, biotin with neurologic toxicity being the dose-limiting factor. After the second operation, median survival was 19 and 11.5 months in patients with anaplastic astrocytoma and glioblastoma, respectively.

Recently, the efficacy of this pretargeting radioimmunotherapy protocol was evaluated in an adjuvant setting (Grana *et al*, 2002). Newly diagnosed patients, 17 with grade III glioma and 20 with glioblastoma, received surgery and external beam radiation. Then, 19 patients received the three reagents in the sequence described above with the ^90^Y-labelled biotin being given at a dose of 2.2 GBq m^−2^, with the remaining 18 patients serving as controls. Unlike the initial clinical study, all reagents were administered via the intravenous route instead of directly into the surgical resection cavity. The median survival estimated for the grade IV glioma patients was 8 months in the control group (*n*=12) and 33.5 months in the treated group (*n*=8). Only two of the 11 treated grade III patients had died at the time of that publication.

The results obtained with this pretargeting protocol are highly encouraging, particularly in light of the fact that significant survival prolongation could be obtained even when then labelled compound was administered intravenously. Furthermore, it was discovered after completion of this trial that the BC-4 mAb hybridoma clone produced an additional nonfunctional light chain (De Santis *et al*, 2003). To solve this problem, a new anti-tenascin mAb, ST2146, has been generated. A multi-centre clinical trial is currently being planned to evaluate the therapeutic potential of pretargeted radioimmunotherapy, with ST2146 mAb providing the tenascin targeting component.

## RADIOLABELLED PEPTIDES

Owing to the infiltrative nature of glioma, methods must be devised for improving the delivery of targeted radiotherapy to tumour cells that are distant from the primary lesion. One of the limitations of mAbs is that they diffuse slowly through tissue as a consequence of their large molecular size. Peptides can have molecular weights two orders of magnitude less than intact mAbs and thus are attractive carrier molecules for the targeted irradiation of distant tumour cells. This strategy is currently being investigated in patients with low-grade gliomas, many of which overexpress somatostatin type 2 receptors (Hofer *et al*, 2001; Schumacher *et al*, 2002).

Five patients with progressive gliomas (two WHO grade II, three WHO grade III) and five patients with surgically debulked WHO grade II gliomas were treated with the labelled somatostatin analogue [^90^Y]-DOTA^0^-D-Phe^1^-Tyr^3^-octreotide. Patients received between one and five cycles of the labelled peptide at a cumulative activity of 555–7030 MBq (15–190 mCi). Responses of 13–45 months duration were observed in the progressive patients. Disease stabilisation was observed in the five newly diagnosed low-grade glioma patients who received radiolabelled peptide therapy following resection. Side effects included increased seizure frequency and were transient. However, the potential clinical role of target radiotherapy in lower grade gliomas is less clear, because a wait-and-see attitude currently prevails for patients with these malignancies.

## CONCLUSIONS AND FUTURE PERSPECTIVES

In summary, clinical trials, primarily with radiolabelled anti-tenascin mAbs, have demonstrated the feasibility of performing targeted radiotherapy in glioma patients. By administering the labelled mAb directly into surgically created resection cavities, a significant survival advantage has been obtained for patients with malignant glioma in comparison with radiation therapy combined with chemotherapy. Furthermore, we have shown that ^131^I–labelled 81C6 results in significantly lower toxicity than other techniques designed to boost radiation dose to the primary tumour site such as stereotactic radiosurgery or brachytherapy.

It remains to be ascertained whether multiple cycles of targeted radiotherapeutic offer a significant survival advantage compared with single-dose protocols. Likewise, it is not clear whether longer range beta emitters such as ^90^Y or shorter range beta emitters such as ^131^I provide the best balance between maximising tumour cell killing and minimising debilitating toxicity to normal brain. The most intriguing challenge for targeted brain tumour radiotherapy will be to develop strategies for delivering curative doses of radiation to small deposits and single glioma cells located centimetres from the primary lesion. Work to date with highly cytotoxic alpha emitters and highly diffusible peptides is encouraging in this regard. Use of micro-infusion techniques such as those being used to treat gliomas with immunotoxins (Nguyen *et al*, 2003) may also play an important role.
